# Sorption of Iodine on Biochar Derived from the Processing of Urban Sludge and Garden Waste at Different Pyrolysis Temperatures

**DOI:** 10.3390/molecules29133007

**Published:** 2024-06-25

**Authors:** Bing Bai, Qingyang Liu, He Li, Dan Liu, Haichao Wang, Chengliang Zhang, Zheng Yang, Jingjing Yao

**Affiliations:** 1 Institute of Resources and Environment, Beijing Academy of Science and Technology, Beijing 100089, China; baibing_1029@163.com (B.B.); whuheli@163.com (H.L.); liujiatong615@163.com (D.L.); wanghaichao9501@163.com (H.W.); zhang64@126.com (C.Z.); 2 College of Ecology and Environment, Nanjing Forestry University, Nanjing 210037, China; 3 Beijing Milu Ecological Research Center, Beijing 100076, China; 3180400016@caa.edu.cn

**Keywords:** sludge waste, garden waste, biochar, iodine

## Abstract

The United Nations proposed the Sustainable Development Goals with the aim to make human settlements in cities resilient and sustainable. The excessive discharge of urban waste including sludge and garden waste can pollute groundwater and lead to the emission of greenhouse gases (e.g., CH_4_). The proper recycling of urban waste is essential for responsible consumption and production, reducing environmental pollution and addressing climate change issues. This study aimed to prepare biochar with high adsorption amounts of iodine using urban sludge and peach wood from garden waste. The study was conducted to examine the variations in the mass ratio between urban sludge and peach wood (2/1, 1/1, and 1/2) as well as pyrolysis temperatures (300 °C, 500 °C, and 700 °C) on the carbon yield and adsorption capacities of biochar. Scanning electron microscopy, Brunauer–Emmett–Teller analysis, Fourier transform infrared spectrometry, powder X-ray diffraction, and elemental analysis were used to characterize the biochar produced at different pyrolysis temperatures and mass ratios. The results indicate that the carbon yield of biochar was found to be the highest (>60%) at a pyrolysis temperature of 300 °C across different pyrolysis temperatures. The absorbed amounts of iodine in the aqueous solution ranged from 86 to 223 mg g^−1^ at a mass ratio of 1:1 between urban sludge and peach wood, which were comparably higher than those observed in other mass ratios. This study advances water treatment by offering a cost-effective method by using biochar derived from the processing of urban sludge and garden waste.

## 1. Introduction

With the acceleration of industrialization and urbanization in China, the production of urban sludge has exhibited an increasing growth trend [1,2]. As of 2020, the annual production of urban sludge is estimated to exceed 60 million tons [3,4]. To address the environmental issues associated with urban sludge, technology in the use of sludge resources has emerged, aiming to reduce environmental risks and generate multiple benefits by recycling and reusing valuable substances in sludge [4,5,6]. Currently, the recycling and reusing rate of sludge treatment in China is approximately 33% nationwide, which is relatively low compared to some areas with high human development [2,7,8]. To improve the recycling and reusing rate of sludge treatment, the “14th Five Year Plan for the Development of Urban Sewage Treatment and Resource Utilization”, jointly issued by the National Development and Reform Commission and the Ministry of Housing and Urban-Rural Development, has set a clear goal of achieving a recycling and reusing rate of sludge treatment of 90% by 2025 [9,10]. Therefore, developing new technologies in achieving the resource utilization, reduction, and treatment of sludge is of crucial significance in promoting the sustainable development of cities in China [3,8].

There are several technologies for the resource utilization of sludge [3,7]. The main pathways for the resource utilization of sludge include sludge fermentation to produce biogas or organic acids [11,12]. However, after the treatment of anaerobic fermentation, the degradation rate of the organic matter in sludge is expected to vary from 40% to 60% [13,14,15]. In other words, the residues of the fermented sludge contain a relatively large amount of organic matter [13]. At present, there is a lack of more effective treatment methods for organic residues after sludge fermentation, apart from landfill or building material utilization [16,17]. Pathogens and toxic metals could pose potentially negative effects on ecosystems through leaching effects when the residues after sludge fermentation are used for landfill or building material utilization [17,18,19].

Pyrolysis technology, as an emerging treatment method, provides new possibilities for the resource utilization of sludge [20]. In anaerobic or hypoxic environments, pyrolysis technology can eliminate harmful substances such as pathogens and fix heavy metals through high-temperature treatment from 300 to 1000 °C [21]. In addition, the pyrolysis process can also produce biochar (solids) and bio-oil (condensed liquids) [22,23]. Pyrolysis mainly includes two main technical paths, which are operated at high pyrolysis temperature and low pyrolysis temperature, respectively [15,23]. Biochar produced in a treated process at a low pyrolysis temperature (<700 °C) could increase the yield of biochar, which has broad application prospects [21,24]. In the 1990s, researchers conducted a study on black soil in the Amazon forest and found that adding biochar could maintain soil fertility for a long time artificially [25,26]. These findings provide multiple applications of biochar in the field of agriculture [25,27]. Biochar is a type of solid material that is produced using the thermochemical conversion of biological solids under oxygen-limited conditions [24]. Due to its excellent pore structure, large surface area, and abundant organic functional groups on the surface, biochar is often used as an adsorbent, soil amendment, and biochar fertilizer [24,25]. The biochar produced from sludge has been successfully used as a soil amendment, which aims to increase the soil pH, enhance soil basal respiration and enzyme activity, and significantly create more favorable conditions for crop growth [23]. A prior study indicated that the biochar based on sludge exhibited an efficient removal effect of hexavalent chromium in soil, further proving its feasibility in land use [28,29].

The adsorption amount of iodine in an aqueous solution is usually the most direct and commonly used indicator to evaluate the adsorption performance of biochar [30,31,32]. Previous studies have shown that the adsorption amounts of iodine by biochar could be used as a metric of activation and product quality for comparisons of biochar across different reaction conditions [31,33]. The preparation of biochar using pure sludge exhibits a relatively low adsorption amount of iodine in an aqueous solution [32]. Thus, the addition of other carbon substances to sludge before the pyrolysis process could improve the adsorption amount of iodine in aqueous solutions by biochar [23,30,32]. The garden waste from urban green spaces is a good source of carbon material, which can be mixed with sludge to prepare biochar with high adsorption amounts of iodine in an aqueous solution [34,35]. The mixtures of sludge with the garden waste from urban green spaces not only improved the performance of biochar in the adsorption performances, but also realized the resource utilization of garden waste [35]. Peach wood is a common garden waste in China, especially in rural areas [11,13,20]. Since peach wood is sensitive to ambient moisture, furniture made of peach wood without drying treatments and fine processing is prone to deformation and cracking [11,13,20]. Therefore, the preparation of biochar with the recycling of fragmented peach wood is a practical way to achieve resource utilization.

This study aimed to prepare biochar with high adsorption capacity using urban sludge and peach wood obtained from garden waste. The study explored different factors including the pyrolysis reaction time, reaction temperature, and mixing ratio on the adsorption capacity of biochar. The characterization of biochar under different reaction conditions was carried out using scanning electron microscopy, Brunauer–Emmett–Teller analysis, Fourier transform infrared spectrometry, powder X-ray diffraction, thermogravimetric analysis, and elemental analysis. The amounts of adsorption capacity in iodine in aqueous solutions across biochar under different reaction conditions were measured and compared to determine the optimization conditions. The potential factors associated with the morphological prosperities, specific surface area, and pore distribution of biochar with high adsorption capacity were discussed. This study provides an efficient and eco-friendly technology for the preparation of biochar using the urban sludge and garden waste from urban green spaces, aiming at the realization of resource utilization.

## 2. Results

### 2.1. Optimal Reaction Conditions

**Figure 1:**
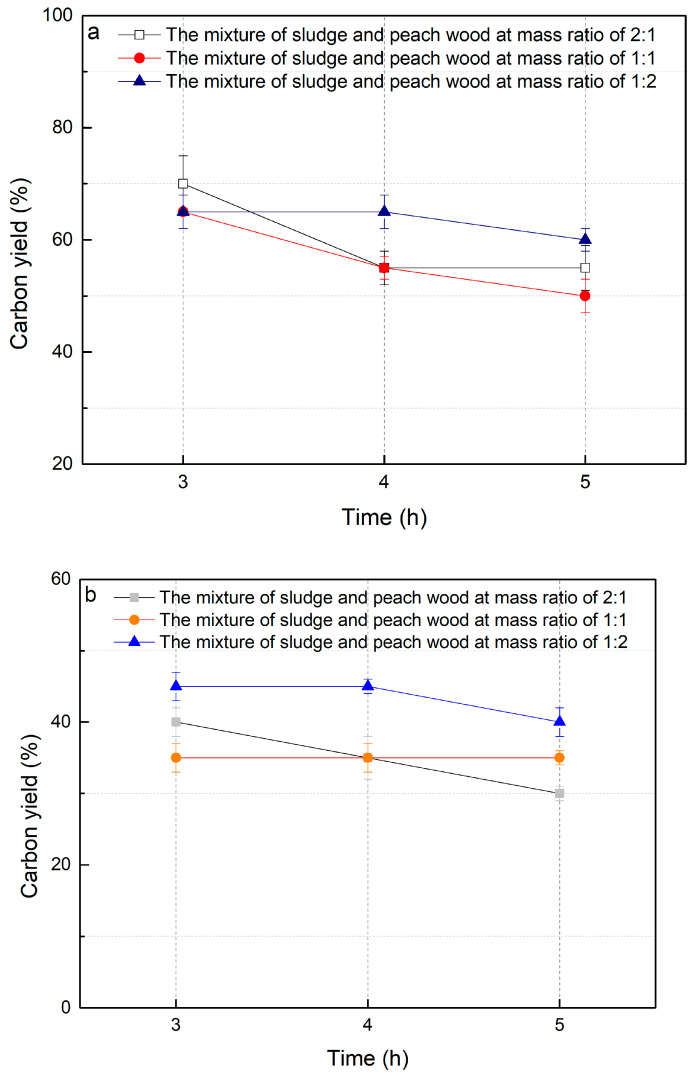

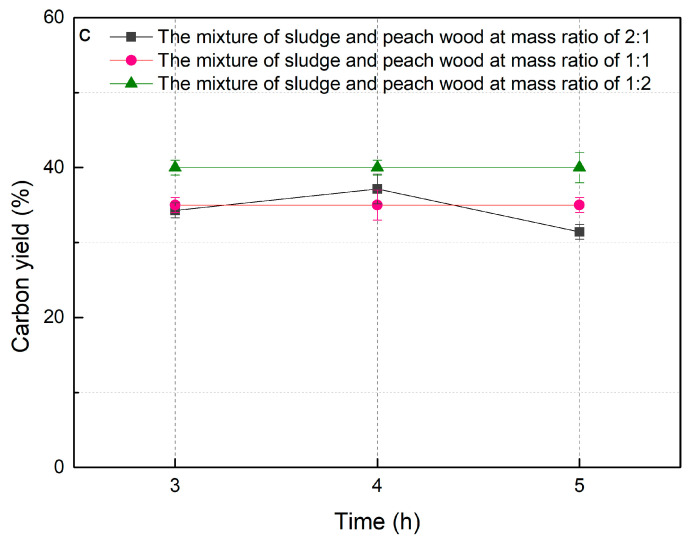
The carbon yield of biochar under different pyrolysis temperatures. (**a**) 300 °C, (**b**) 500 °C, (**c**) 700 °C.

Table 1 presents the elemental composition (C, O, N, S, and H) and ash content of sludge and peach wood. The carbon content of sludge and peach wood were found to be 18% and 50%, respectively. The reaction temperature range from 300 °C to 700 °C was selected because this temperature range is an appropriate condition for the formation of biochar under pyrolysis [17,20,21]. Lower temperatures cannot lead to the carbonization of raw materials, while higher temperatures result in changes to the biochar properties [17,20,21]. Prior studies found that the preparation of biochar at temperatures higher than 700 °C could produce porous materials with higher surface areas but a lower content of carbon yield relative to those at temperatures lower than 700 °C [17,20,21]. A greater loss of carbon during the preparation of biochar at temperatures higher than 700 °C may release excess CO_2_ emissions. Considering the potential environmental impacts, this study attempted to choose an optimal temperature range from 300 °C to 700 °C [17,20,21]. Within the optimal temperature range, this study selected the initial temperature (300 °C), the median temperature (500 °C), and the final temperature (700 °C) of the temperature range. The as-made biochar was collected after the materials were cooled to room temperature inside the furnace. From Figure 1, it can be seen that under the same mass ratio between sludge and peach wood as well as the reaction time, the carbon yield did not necessarily increase with the increase in temperature. Under the condition of a sludge to peach wood ratio of 2:1 and reaction time of 3 h, the carbon yield was 34% at 700 °C, 40% at 500 °C, and as high as 70% at 300 °C. 

This result indicates that the relationship between the yield of biochar and the reaction temperature is not linear. For the conditions of the sludge to peach wood ratios of 2:1 and 1:1, the carbon yields of biochar at 300 °C were observed to be higher than those at 500 °C and 700 °C, respectively. This finding indicates that the optimal reaction temperature in this study was found to be 300 °C.

At the same reaction temperature and time, the ratio of sludge to peach wood did not have a significant impact on the charcoal yield. For most cases, the variation in the proportion did not significantly change the carbon yield. When the reaction temperature was 300 °C and the reaction time was 3 h, the average carbon yield of biochar was 65% using a sludge to peach wood ratio of 1:2, which was slightly higher than those in other conditions. The lowest average carbon yield of biochar was 34% under the conditions of a reaction temperature of 700 °C and a reaction time of 3 h.

As seen in Figure 1, it was found that the increase in reaction time did not significantly improve the carbon yield. This result may be ascribed to the higher reaction temperature possibly leading to the oxidation of carbon to form CO_2_ in biochar [36]. In this study, the optimal reaction temperature was a dominant factor for the carbon yield of biochar relative to the other factors including the mixing ratio and reaction time.

### 2.2. Physicochemical Properties of Biochar

#### 2.2.1. Specific Surface Area and Total Pore Volume

**Figure 2:**
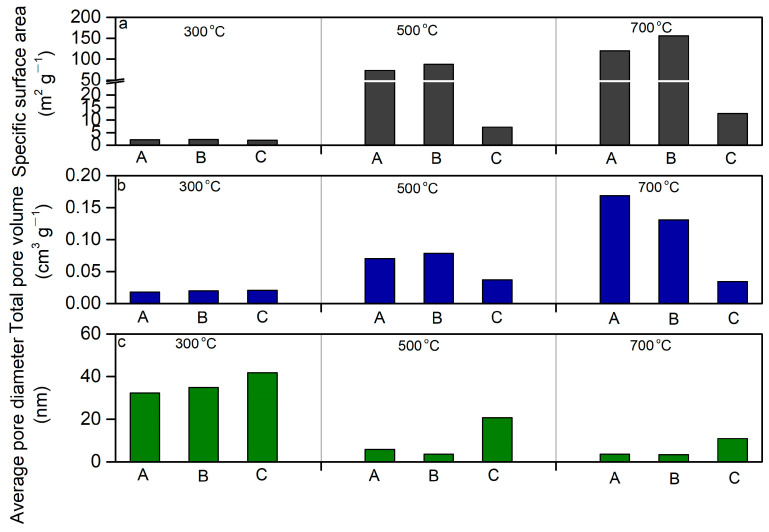
The specific surface area (**a**), total pore volume (**b**), and average pore diameter of biochar (**c**) under different pyrolysis temperatures. A—The mixture of sludge and peach wood at a mass ratio of 2:1. B—The mixture of sludge and peach wood at a mass ratio of 1:1. C—The mixture of sludge and peach wood at a mass ratio of 1:2.

At a reaction temperature of 300 °C, the levels of specific surface area were generally low, ranging from 2.22 to 2.27 m^2^ g^−^^1^ (Figure 2a). When the reaction temperature was set to 500 °C, the levels of the specific surface area increased remarkably (from 73.22 to 87.72 m^2^ g^−^^1^). With the reaction temperature of 700 °C, the greatest level of specific surface area (150.0 m^2^ g^−^^1^) in biochar at a mass ratio of 1:1 was observed (Figure 2). Similar trends in the total pore volume associated with the reaction temperature were found. The average level of the total pore volume varied from 0.02 to 0.04 cm^3^ g^−^^1^ in the biochar at a reaction temperature of 300 °C. The average level of the total pore volume was in the range of 0.04 to 0.10 cm^3^ g^−^^1^ in the biochar at a reaction temperature of 300 °C. The highest level of total pore volume was 0.15 cm^3^ g^−^^1^ at a reaction temperature of 700 °C in the biochar at a mass ratio of 1:1 (Figure 2b). In contrast, the mixing ratio and reaction time had minor impacts on the levels of specific surface area and total pore volume in the biochar under the same reaction temperature.

#### 2.2.2. Pore Diameter

Contrary to the trends of specific surface area and total pore volume against the reaction temperature, the average pore diameter of biochar at a reaction of 300 °C was found to be larger than those observed at 500 °C and 700 °C, respectively. The average pore diameter of biochar at a reaction of 300 °C ranged from 25 to 40 nm, while the average pore diameter of biochar was in the range of 10–24 nm and 6–12 nm, respectively (Figure 2c). The results indicate that higher temperatures could lead to structure damage of the wood, thus decreasing the pore diameter of biochar. The enhancement in the reaction temperature could change the fracture pore structure of biochar to a complex composition, resulting in increases in the specific surface area and total pore volume [37].

**Figure 3:**
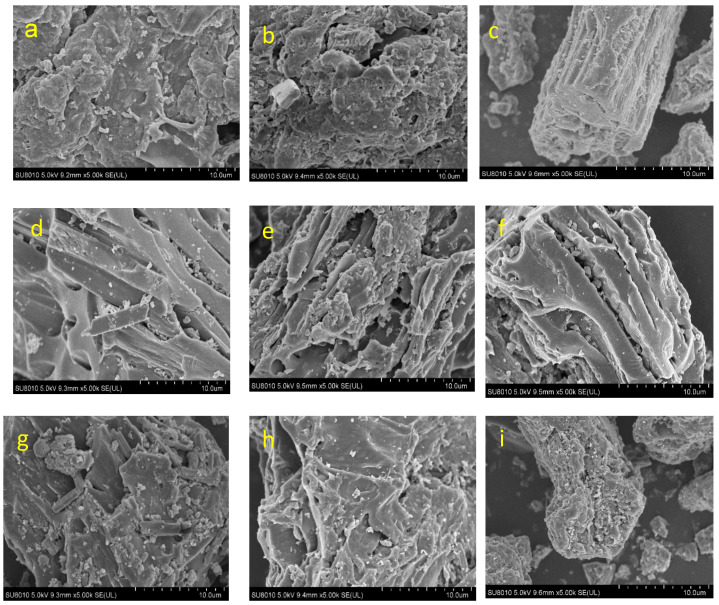
Scanning electron microscope images of biochar under different conditions. The accelerating voltage, 15 kV. (**a**) The mixture of sludge and peach wood at a mass ratio of 2:1 under 300 °C. (**b**) The mixture of sludge and peach wood at a mass ratio of 1:1 under 300 °C. (**c**) The mixture of sludge and peach wood at a mass ratio of 1:2 under 300 °C. (**d**) The mixture of sludge and peach wood at a mass ratio of 2:1 under 500 °C. (**e**) The mixture of sludge and peach wood at a mass ratio of 1:1 under 500 °C. (**f**) The mixture of sludge and peach wood at a mass ratio of 1:2 under 500 °C. (**g**) The mixture of sludge and peach wood at a mass ratio of 2:1 under 700 °C. (**h**) The mixture of sludge and peach wood at a mass ratio of 1:1 under 700 °C. (**i**) The mixture of sludge and peach wood at a mass ratio of 1:2 under 700 °C.

We used a scanning electron microscope to observe the structural characteristics of the biochar produced at different temperatures (Figure 3). Usually, biochar exhibits an abundant and diverse pore structure on its surface after pyrolysis, providing good adsorption performance [38,39]. The pyrolysis of sludge could generate fracture pores, which are generally irregular [17]. This irregularity could result from the complex composition of sludge and the instability of the pore structure associated with the interaction between different components during the pyrolysis process [15]. In contrast, the relatively regular honeycomb-arranged tubular pores could be ascribed to the structure of the peach wood chips after pyrolysis [32]. With increases in the pyrolysis temperature, the existence of these tubular pores not only increases the specific surface area of the biochar, but also facilitates the diffusion and transmission of substances within the pores [24]. The differences in the total pore volume of biochar produced from different pyrolysis temperatures were not observed clearly using a scanning electron microscope. However, the levels in the total pore volume of the biochar across different pyrolysis temperatures were determined by Brunauer–Emmett–Teller analysis (Figure 2).

#### 2.2.3. pH

**Figure 4:**
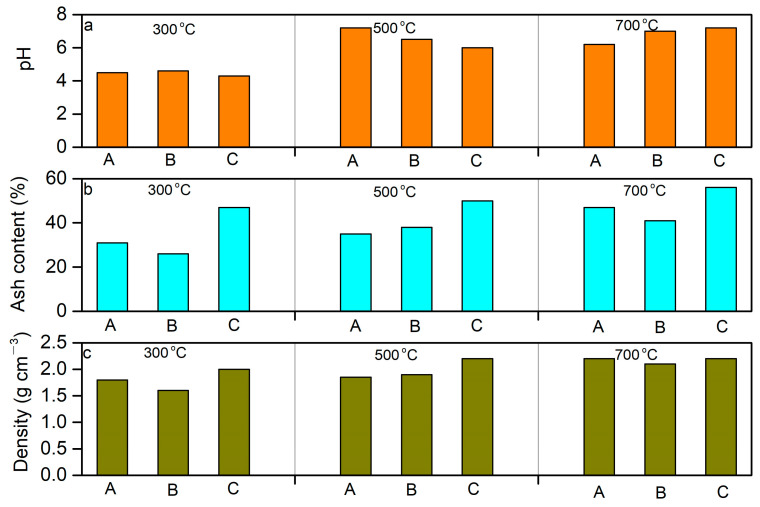
The pH (**a**), ash content (**b**), and density (**c**) of biochar under different pyrolysis temperatures. A—The mixture of sludge and peach wood at a mass ratio of 2:1. B—The mixture of sludge and peach wood at a mass ratio of 1:1. C—The mixture of sludge and peach wood at a mass ratio of 1:2.

At a reaction temperature of 300 °C, the surface of the biochar exhibited acidity, with pH values in the range from 4.2 to 4.5. When the reaction temperature increased to 500 °C, the surface of the biochar became less acidic, with pH values varying from 5.4 to 7.1. At a reaction temperature of 700 °C, the surfaces of the biochar were neutral, with pH values from 6.2 to 7.4 (Figure 4a). The results derived from the pH values of the biochar support prior findings that indicated that the biochar of wood resulted from the higher temperature neutralizing the free acids in sludge [11,14,15]. 

At a mass mixing ratio of 2:1 (sludge/peach wood) at different reaction temperatures, the pH values showed large variations from 4.4 to 7.1. At a mass mixing ratio of 1:2 (sludge/peach wood) at different reaction temperatures, the pH values were found to be in the range of 4.2 to 7.5, presenting obvious variations. In contrast, the pH values were relatively constant at a mass mixing ratio of 1:1 (sludge/peach wood), ranging from 4.5 to 6.9. The results were consistent with the prior findings that the biochar of wood can neutralize free acids in sludge. Therefore, the biochar produced from equal mass proportions of sludge and peach wood showed relatively slight variations in pH values compared to those derived from unequal mass proportions. 

#### 2.2.4. Ash Content

The ash contents were observed to vary from 26% to 31% at a reaction temperature of 300 °C, which were comparably lower than those obtained at reaction temperatures of 500 °C and 700 °C, respectively. The ash contents at a reaction temperature of 500 °C and 700 °C ranged from 35% to 38% and 41% to 56%, respectively (Figure 4b). The increases in the ash contents of biochar with increasing reaction temperature proved that higher temperatures could lead to the relatively high carbon loss of peach wood and sludge, resulting in the higher ash content and lower carbon yield of biochar.

At a mass mixing ratio of 2:1 (sludge/peach wood), the ash contents were found to be in the range of 31% to 47%, while the ash content was from 26% to 42% at a mass mixing ratio of 1:1 (sludge/peach wood). The ash contents of biochar were found to be higher at a mass mixing ratio of 1:2 (sludge/peach wood), which varied from 50% to 56%. These results indicate that the introduction of peach wood could lead to the production of ash contents at a relatively higher temperature.

#### 2.2.5. Density

Density is an important physical property of biochar because its packing density can influence the transportation and storage performance depending on the application [39]. The density of biochar obtained at a reaction temperature of 300 °C was found to vary from 1.6 g cm^−^^3^ to 1.8 g cm^−^^3^, which was comparably lower to those obtained at reaction temperatures of 500 °C (1.8–1.9 g cm^−^^3^) and 700 °C (2.2 g cm^−^^3^), respectively (Figure 4c). At a mass mixing ratio of 2:1 (sludge/peach wood), the density of biochar was in the range of 2.0–2.3 g cm^−^^3^, which was slightly greater than those at a mass ratio of 1:1 (1.6–2.0 g cm^−^^3^) and 1:2 (1.7–1.8 g cm^−^^3^) between sludge and peach wood, respectively. The trends in the density of biochar were consistent with those observed in the ash content, which showed that the addition of peach wood could enhance the density of biochar, while the higher temperature could lead to the carbon loss in peach wood, and thus decrease the density of biochar. Our results show that the reaction temperature and mass proportion of peach wood are the main factors influencing the density of biochar. Thus, it is necessary to adjust the reaction conditions (e.g., reaction temperature, mass proportion) to obtain biochar with the appropriate density according to specific needs in the production and application of biochar [35].

#### 2.2.6. Elemental Composition

**Figure 5:**
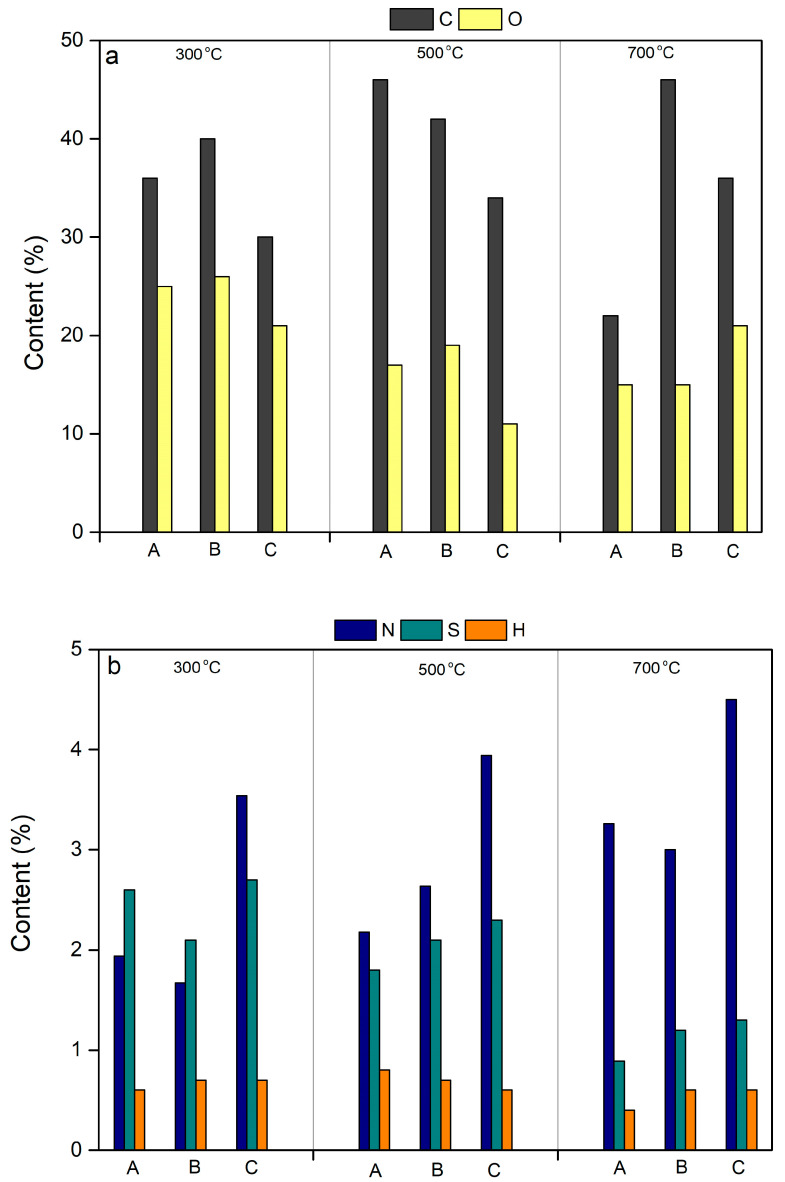
The contents of the elemental composition including carbon (**a**), oxygen (**a**), sulfur (**b**), nitrogen (**b**), and hydrogen (**b**) under different pyrolysis temperatures. A—The mixture of sludge and peach wood at a mass ratio of 2:1. B—The mixture of sludge and peach wood at a mass ratio of 1:1. C—The mixture of sludge and peach wood at a mass ratio of 1:2.

The contents of C, O, N, S, and H in biochar obtained at different reaction temperatures were found to be in the range of 20–45%, 12–27%, 1.8–4.0%, 0.8–2.7%, and 0.3–0.5%, respectively (Figure 5). The total contents of C, O, N, S, and H in the biochar were observed to vary from approximately 70% to 85%. The undetected contents by elemental composition in the biochar were considered to be associated with mineral elements including Si, Al, Fe, etc. [23]. We performed an analysis of the inorganic substances using powder X-ray diffraction. The presence of other metals was undetected using powder X-ray diffraction because the contents of these metals were at the trace level (~ppt) and below the detection limits of the powder X-ray diffraction (~ppm) [23]. The elemental composition contents of C and O in the biochar were higher than those in sludge but lower than those in peach wood, while the contents of N and S in the biochar were lower than those in sludge but greater than those in peach wood. The contents of H in biochar were lower than those found in sludge and peach wood (Table 1). These findings indicate that the preparation of biochar can increase the bio-safety disposal of biochar by fixing the environmental component of hazardous substances (e.g., N and S) [21]. In addition, the preparation of biochar did not influence the combustion energy density compared to the peach wood because comparable levels of carbon content were found between biochar and peach wood. As seen in Figure 1, high ratios of H/C were observed in biochar obtained at different reaction temperatures (i.e., 300 °C, 500 °C, and 700 °C) relative to those in peach wood and sludge, featuring the formations of aromatic compounds on the surfaces of biochar [24].

**Figure 6:**
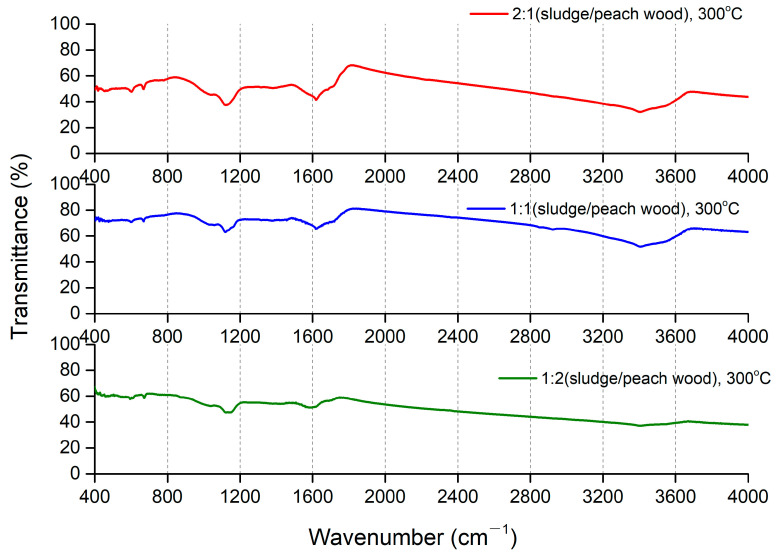
Fourier transform infrared spectrometer spectra of biochar at a pyrolysis temperature of 300 °C.

Through FTIR analysis, we observed the characteristic peaks of different functional groups (Figure 6, Figures S2 and S3) including the peaks that appeared at 3414 cm^−^^1^ and 3600 cm^−^^1^. The existence of the peak at 3414 cm^−^^1^ could be attributed to the vibration of the C-OH bond [40], while the presence of 3600 cm^−^^1^ could be linked with the adsorbed water [38]. The peaks at 2900 cm^−^^1^ and 600–700 cm^−^^1^ demonstrated the stretching and bending vibration of the C-H bond, respectively [38]. The peaks indicated the presence of alcohols or phenolic compounds in the biochar. The peak at ~1600 cm^−^^1^ belongs to the vibration of C=C, indicating the presence of olefins or aromatic structures in the biochar [39]. The peaks 1100–1200 cm^−^^1^ were attributed to the vibration of the C-O bond [38]. In addition, the peak shown at ~1042 cm^−^^1^ is a vibration of C-O-C, indicating that the ether compounds were on the surface of the biochar. Notably, we observed the presence at 1400 cm^−^^1^ in biochar produced at 700 °C with the mixture of sludge and peach wood at a mass ratio of 1:2 (Figure S3). The peak at 1400 cm^−^^1^ could be associated with the vibration of the C-H bond. The existence of C-H could be explained by the unequal amounts of wood generated by new products due to the greater loss of moisture contents under higher temperatures (700 °C) compared to those under lower temperatures (300 °C and 500 °C). The peaks associated with the vibration of C-N and C-S were not observed via FTIR analysis, indicating that the contents of N and S were not heteroatoms in carbon structures in biochar. The contents of adsorbed water were estimated using the intensities of the O-H line at 3400 cm^−^^1^. We did not find that the amount of adsorbed water correlated with the porosity and adsorption capabilities of biochar produced at different temperatures. In addition, we did not find an association between the intensities of the C=O line at 1600 cm^−^^1^ and oxygen amounts measured by the elemental composition. This result may be explained by the fact that the contents of the bonded oxygen were measured by FIIR and the total contents of oxygen were determined by the elemental composition. After comparing the peak intensities of the functional groups in biochar across different reaction conditions using FTIR, the levels of intensities from the same functional groups in biochar across different reaction conditions were similar, indicating comparable abundances in the aromatic compounds on the surfaces of the biochar [21]. 

**Figure 7:**
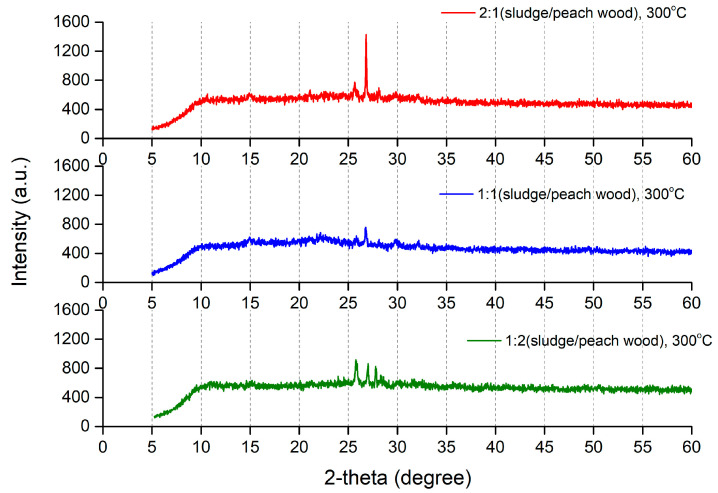
Powder X-ray diffraction diffractograms of biochar at the pyrolysis temperature of 300 °C.

As shown in Figure 7, all of the prepared biochar had a peak at ~25 degrees, which corresponded to the 0.34 nm (graphite (002)) interplanar distance (Figure 7, Figures S4 and S5). This peak is typical of all amorphous carbon structures, indicating the presence of biochar [41,42]. Moreover, the biochar exhibited a strong peak of around 29 degrees (2-theta), which could be ascribed to the presence of carbonates derived from the sludge [17]. When the pyrolysis temperature was increased to 700 °C, the biochar showed a peak at ~35 degrees (2-theta), which could be associated with the formation of carbonates originating from sludge [17,41,42]. Although impurity peaks of some metallic elements were observed in the biochar, as seen in Figure 7, the peak intensities were observed to be relatively low, indicating that the levels of metallic elements were minor [23]. The findings from the XRD analysis indicate that the released amounts of toxic bioavailable metals in the biochar obtained from sludge and peach wood in this study were considered to be negligible.

### 2.3. Adsorption Experiment

In this study, we compared the adsorption performance of the biochar using iodine ions because the adsorption amounts of iodine are widely used as indicators to assess the adsorption capacities of biochar [30,31,32]. At a mass mixing ratio of 2:1 (sludge/peach wood), the amount of adsorbed iodine in the aqueous solution was found to be 32.9 mg g^−^^1^ at the reaction temperature of 300 °C (Table 2). The amount of adsorbed iodine peaked at 63.4 mg g^−^^1^ at the reaction temperature of 500 °C, and then decreased to 40.6 mg g^−^^1^ at the reaction temperature of 700 °C. The amount of adsorbed iodine was observed to be higher at 500 °C than 300 °C, which could be ascribed to the greater total pore volume and specific surface area found at 500 °C at a mass mixing ratio of 2:1 (sludge/peach wood) (Figure 2a,b). The amount of adsorbed iodine decreased at 700 °C relative to 500 °C because a relatively lower average pore diameter was found at 700 °C. Prior studies have indicated that iodine ions could be adsorbed on the surfaces of the biochar through the formation of sp^2^-π type hydrogen bonding [15,16,17]. Higher levels of average pore diameter and specific surface area may generate more active sites for bonding with iodine ions. As shown in Table 2, the amount of absorbed iodine ions varied largely with the pH levels of biochar at similar levels of pH. 

The amounts of adsorbed iodine were highest (223.3 mg g^−^^1^) at a reaction temperature of 300 °C, followed by 131.9 mg g^−^^1^ at a reaction temperature of 500 °C, and 86.3 mg g^−^^1^ at reaction temperature of 700 °C, when the sludge and peach wood was mixed at a mass ratio of 1:1. A similar trend in the amounts of adsorbed iodine dependent on the decreases in the reaction temperature was also observed when the mass mixing ratio between the sludge and peach wood was 1:2. The greatest amounts of adsorbed iodine were 78.6 mg g^−^^1^ at a reaction temperature of 300 °C, which was higher than those at reaction temperatures of 500 °C (74.2 mg g^−^^1^) and 700 °C (55.8 mg g^−^^1^), respectively. At the same reaction temperature, higher amounts of adsorbed iodine were found at a mass mixing ratio of 1:1 (sludge/peach wood), which was consistent with the trends in the average pore diameter across different mass mixing ratios between the sludge and peach wood. Our prepared biochar exhibited excellent performance in the amount of adsorbed iodine compared to several prior studies [30,31,33]. The findings from the adsorbed experiment showed that the mixture of sludge and peach wood, prepared at a mass ratio of 1:1 at a lower reaction temperature (300 °C), could lead to the formation of a relatively high average pore diameter, aiding in the absorption of greater amounts of iodine in aqueous solution. However, this study had some limitations. The adsorption mechanism between iodine ions and biochar was not illustrated using Raman spectroscopy. Previous studies have shown the occurrence of the electron-donating effect and π–π interactions between amorphous carbon structures and ions with sp^2^ bonds [43]. Our adsorption results are consistent with prior studies. This study did not assess the co-existing hazardous contaminants and water-soluble ions on the adsorption capacities of the studied biochar. In addition, the adsorption capacities of iodine ions in real-world samples using the studied biochar were not evaluated. Further studies can address these challenges for the industrial application of the studied biochar. 

## 3. Materials and Methods

### 3.1. Preparation of Biochar

The sludge was collected from a waste treatment plant in Beijing, and the peach wood was taken from the Changping Experimental Base of the Beijing Institute of Resources and Environment (Beijing, China). Detailed quantitative analyses including the content (%) of the elemental compositions (C, O, N, S, and H) and ash were performed for the sludge and peach wood, respectively (Table 1). The sludge and peach wood chips were mixed at a mass ratio of 2:1, 1:1, and 1:2, respectively. Then, the mixtures were finely crushed into particles with a diameter of 1 cm and a length of 2 cm using a compression granulator. Subsequently, the mixtures were placed in a pyrolysis carbonization furnace for pyrolysis under a nitrogen atmosphere at a heating rate of 10 °C min^−^^1^ for different periods (i.e., 3, 4, and 5 h). After cooling to room temperature inside the furnace, the chunk biochar was collected and crushed into particles. The crushed particles were passed through a 200-mesh sieve and washed with 75% of alcohol solution several times to remove possible contaminants. The products were then dried at 80 °C for 12 h and stored for further use [29].

### 3.2. Analysis of Biochar

The yield of biochar was estimated using Formula (1):

(1) $$W=\frac{M_{2}}{M_{1}}\times 100\%$$

where W represents the yield of biochar, in%; M_1_ is the original mass of the raw material in grams; M_2_ is the mass of biochar after pyrolysis of the raw material in grams [36].

The ash content of the biochar was carried out using a previously reported method [44]. Briefly, 1.0 g of biochar was accurately weighed in a porcelain crucible. Then, the samples were heated at 800 ± 20 °C for 2 h. After the samples were cooled for 5 min, the samples were placed in a dryer and cooled to room temperature. Subsequently, the burned samples were weighed. The ash content of biochar was calculated using Formula (2):

(2) $$A=\frac{M_{2}}{M_{1}}\times 100\%$$

where A refers to the ash content (%); M_2_ refers to the mass of biochar burned after constant weight in grams; M_1_ refers to the amount of biochar in grams.

Accurate 4.0 g biochar samples were placed in a 10 mL centrifuge tube and mixed with 8 mL of distilled water. At the room temperature of 25 °C, the centrifuge tube was shaken for 24 h by a rotation table. Then, the pH of the supernatant solution was measured using a pH meter [45].

The density of biochar under different reaction conditions was performed using a density bottle [46]. Accurate 2.0 g biochar samples in a density bottle mixed with a solution of ethanol were placed in a water bath at a constant temperature (20 °C). Then, the mixture mass of the biochar and density bottle were measured. The density of the biochar was estimated by Formula (3):

(3) $$D=\frac{M_{1}-M_{0}}{V}$$

where D is the density in g cm^−3^; M_1_ is the mass of the density bottle with biochar added in grams; M_0_ is the mass of the density bottle; V is the volume of the density bottle in cm^−3^.

### 3.3. Characterization of Biochar

The biochar was first dispersed on the surface of copper columns containing conductive adhesive and then treated with spray-gold. The morphological prosperities of the biochar were observed using scanning electron microscopy (BEL Japan, Inc., Toyonaka, Japan) [47]. The accelerating voltage was set to 15 kV. The specific surface area and pore distribution of the biochar were measured by Brunauer–Emmett–Teller analysis (Figure S1) with a MICROMERITICS 3 FLEX instrument (Norcross, GA, USA) at a degassing temperature of 77 K [48]. We recorded the Fourier transform infrared (FTIR) spectra of the biochar at different reaction conditions using a Bruker VERTEX 80 V (Billerica, MA, USA) [40]. The biochar was deposited on KBr pellets and observed from 4000 to 450 cm^−1^ with a resolution of 0.2 cm^−1^. Powder X-ray diffraction (XRD) was used to characterize the crystalline structures and phase composition of biochar at different reaction conditions, which was measured by a Rigaku Ultima IV X-ray diffractometer (Tokyo, Japan) with Cu Kα radiation at 1.54 Å [49]. The contents of elements including C, O, N, S, and H were measured using a vario El cube elemental analyzer (Elemntar, Analysensysteme GmbH, Frankfurt, Germany) coupled to a thermal conductivity detector [50].

### 3.4. Batch Experiment

The amounts of adsorbed iodine in aqueous solution were determined using a prior method [31,51]. In brief, an accurate 0.5 g of the biochar sample was put in a ground conical flask and mixed with 50 mL of 0.1 mol L^−1^ iodine solution. Then, the solution was mixed for 15 min. Subsequently, 10 mL of the supernatant solution was taken out and mixed with 50 mL of distilled water and 2 mL of the starch indicator. Then, 0.1 mol L^−1^ of sodium thiosulfate standard solution was added to the supernatant solution in a dropwise manner. The iodine in the supernatant solution reacts with the sodium thiosulfate solution to form a complex, resulting in the solution being blue. After the volume of sodium thiosulfate standard was recorded, the amounts of adsorbed iodine in aqueous solution were calculated according to the following formula:

(4) $$A=\frac{\left(5\times (10{\times N}_{1}-V\times N_{2})\times 127\right)}{M}$$

where M is the mass of the char sample to be tested in grams; V is the volume of standard sodium thiosulfate solution consumed in mL; N_2_ is the concentration of sodium thiosulfate in a sodium thiosulfate solution measured in mol L^−1^; N_1_ is the concentration of iodine in iodine solution measured in mol L^−1^; A is the iodine adsorption value in mg g^−1^.

## 4. Conclusions

In conclusion, our study demonstrated that biochar with high adsorption capacities of iodine could be successfully prepared using urban sludge and garden waste under pyrolysis conditions. We used the combination of urban sludge and peach wood to produce biochar because peach wood can provide abundant sources of carbon, and sludge can maintain the tubular pores and higher specific surface area of biochar at pyrolysis temperatures. The composite structure could enhance the adsorption properties of biochar. We investigated the different parameters (e.g., the mass ratio between urban sludge and peach wood, pyrolysis temperatures) on the carbon yield and adsorption capacities of the biochar. The optimal condition was conducted at a pyrolysis temperature of 300 °C using a mass ratio of 1:1 sludge and peach wood. Under the optimal conditions, the carbon yield of biochar was found to be higher than 60% with the specific surface area, total pore volume, and average pore diameter in the range of 2–5 m^2^ g^−1^, 0.02–0.05 cm^3^ g^−1^, and 25–45 nm, respectively. The highest amount of iodine in the aqueous solution by biochar was observed to be 223.3 mg g^−1^, which was prepared at a pyrolysis temperature of 300 °C using a mass ratio of 1:1 sludge and peach wood. This study made efforts to optimize biochar application with the use of urban waste for water management.

## Figures and Tables

**Table 1:** The elemental composition (C, O, N, S, and H) and ash content (%) of sludge and peach wood for the preparation of biochar under different pyrolysis temperatures.

Item	C (%)	O (%)	N (%)	S (%)	H (%)	Ash Content (%)
Sludge	18	21	3	4	1	39
Peach wood	50	45	0.1	n.d.	5	6

n.d.: Lower than the detection limit (0.1%).

**Table 2:** The amounts of adsorbed iodine in aqueous solution by biochar using the mixtures of sludge and peach wood under different pyrolysis temperatures.

Pyrolysis Temperature (°C)	Mass Ratio between Sludge and Peach Wood	Amounts (mg g^−1^)
	2:1	32.9
300	1:1	223.3
	1:2	78.6
	2:1	63.4
500	1:1	131.9
	1:2	74.2
	2:1	40.6
700	1:1	86.3
	1:2	55.8

## Data Availability

Data are contained within the article and Supplementary Materials.

## Supplementary Materials

The following supporting information can be downloaded at: https://www.mdpi.com/article/10.3390/molecules29133007/s1, Figure S1: The adsorption and desorption curves from BET analysis under N_2_ atmosphere; Figure S2: Fourier transform infrared spectrometer spectra of biochars at pyrolysis temperature of 500 °C; Figure S3: Fourier transform infrared spectrometer spectra of biochars at pyrolysis temperature of 700 °C; Figure S4: Powder X-ray diffraction diffractograms of biochars at pyrolysis temperature of 500 °C; Figure S5: Powder X-ray diffraction diffractograms of biochars at pyrolysis temperature of 700 °C.
