# Supplementary material for: Sorption of Iodine on Biochar Derived from the Processing of Urban Sludge and Garden Waste at Different Pyrolysis Temperatures
Source: Molecules. 2024 Jun 25;29(13):3007. doi: 10.3390/molecules29133007 (PMC11243037; doi:10.3390/molecules29133007)
Supplement: Supplementary file 1 [file molecules-29-03007-s001.zip › molecules-3073836-supplementary.pdf]

molecules

Supplementary Materials

Sorption of Iodine on Biochar Derived from the Processing of Urban Sludge and Garden Waste at Different Pyrolysis Temperatures

Bing Bai [1], Qingyang Liu [1,2,*], He Li [1], Dan Liu [1], Haichao Wang [1], Chengliang Zhang [1], Zheng Yang [3] and Jingjing Yao [1,*]

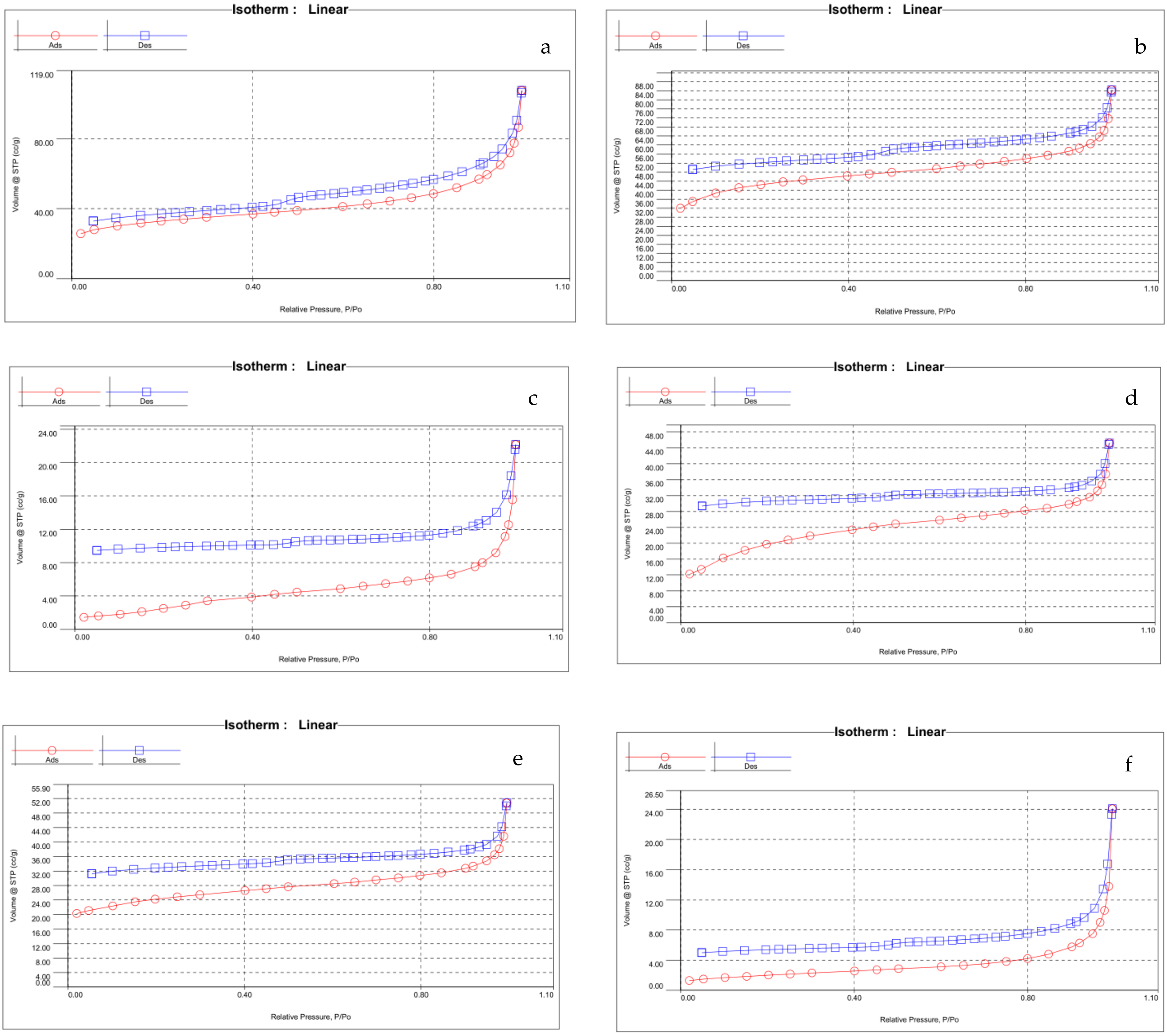

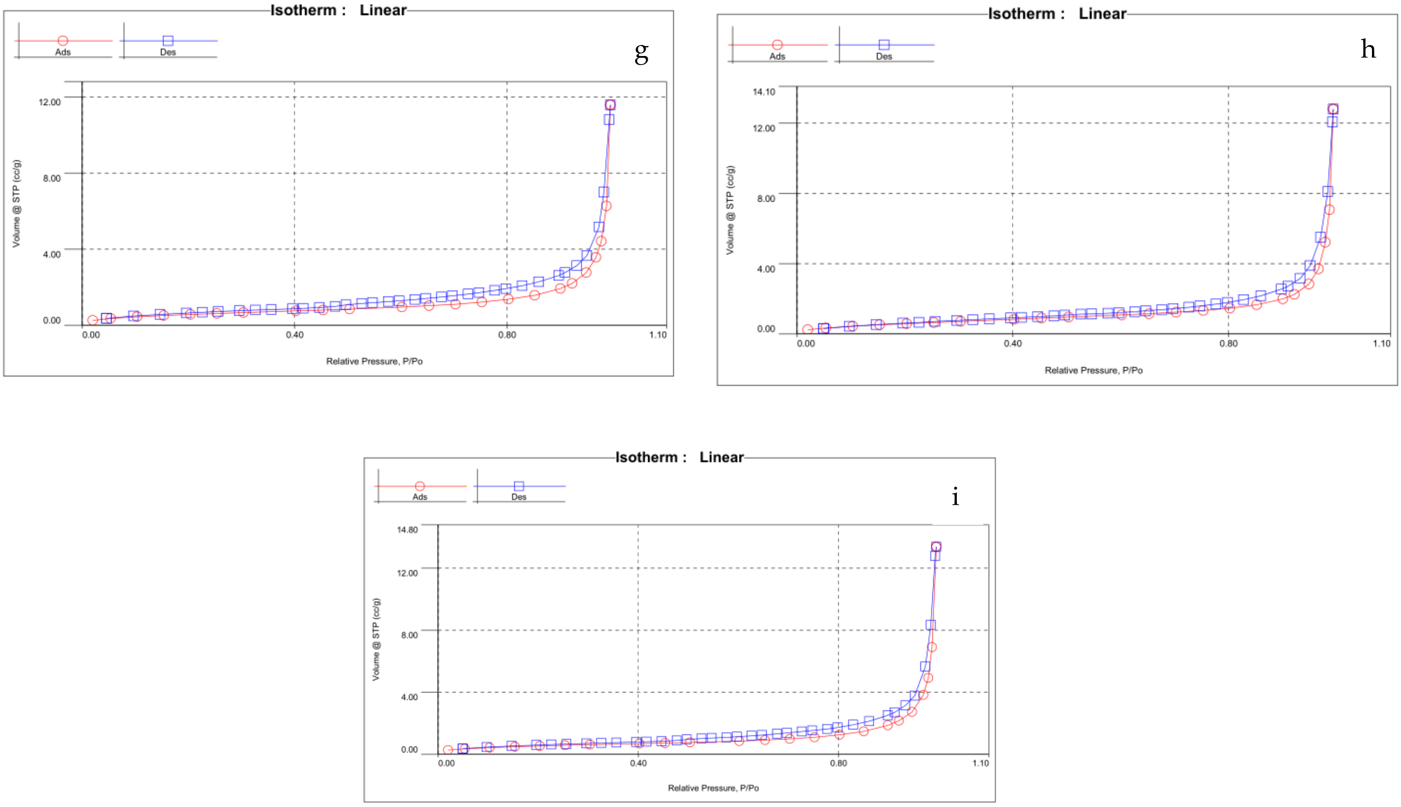

Figure S1. The adsorption and desorption curves from BET analysis under N_2 atmosphere. a. The mixture of sludge and peach wood at a mass ratio of 2:1 under 300°C. b. The mixture of sludge and peach wood at a mass ratio of 1:1 under 300°C. C. The mixture of sludge and peach wood at a mass ratio of 1:2 under 300°C. d. The mixture of sludge and peach wood at a mass ratio of 2:1 under 500°C. e. The mixture of sludge and peach wood at a mass ratio of 1:1 under 500°C. f. The mixture of sludge and peach wood at a mass ratio of 1:2 under 500°C. g. The mixture of sludge and peach wood at a mass ratio of 2:1 under 700°C. h. The mixture of sludge and peach wood at a mass ratio of 1:1 under 700°C. i. The mixture of sludge and peach wood at a mass ratio of 1:2 under 700°C.

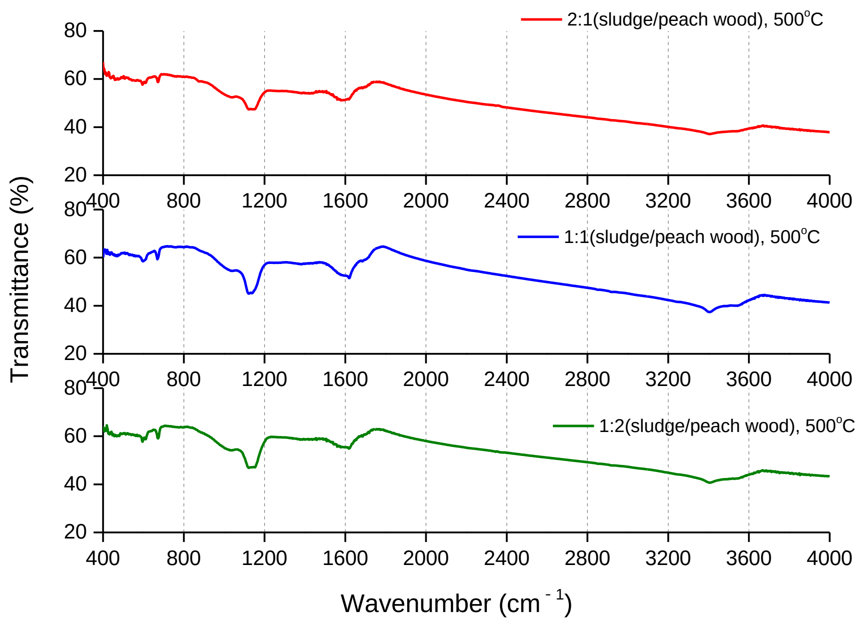

Figure S2. Fourier transform infrared spectrometerspectra of biocharsat pyrolysis temperature of 500℃.

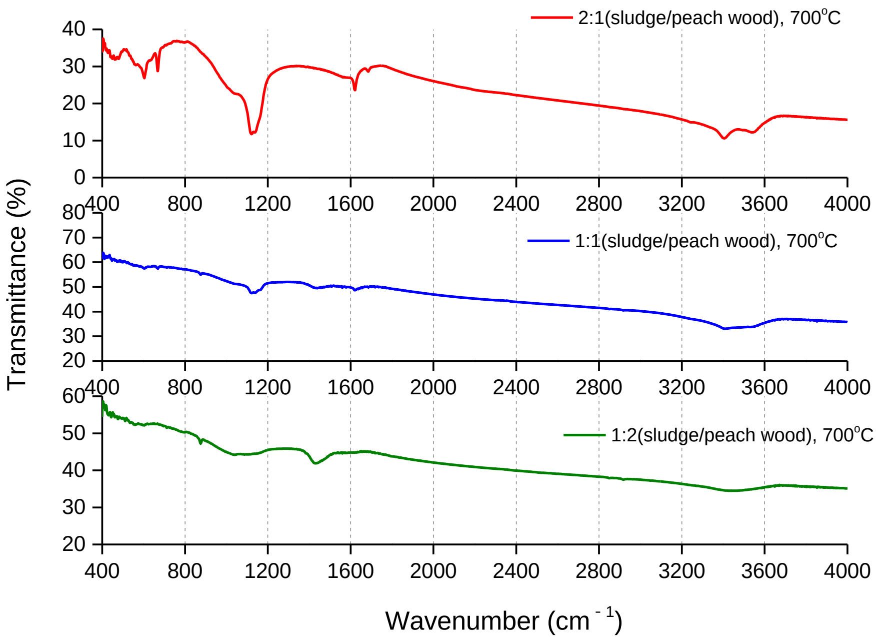

Figure S3. Fourier transform infrared spectrometerspectra of biocharsat pyrolysis temperature of 700℃.

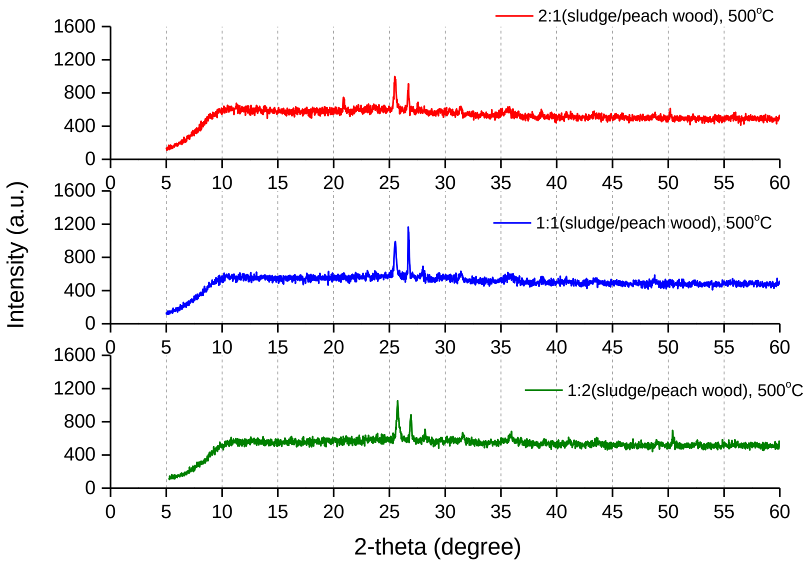

Figure S4.Powder X-ray diffraction diffractograms of biocharsat pyrolysis temperature of 500°C.

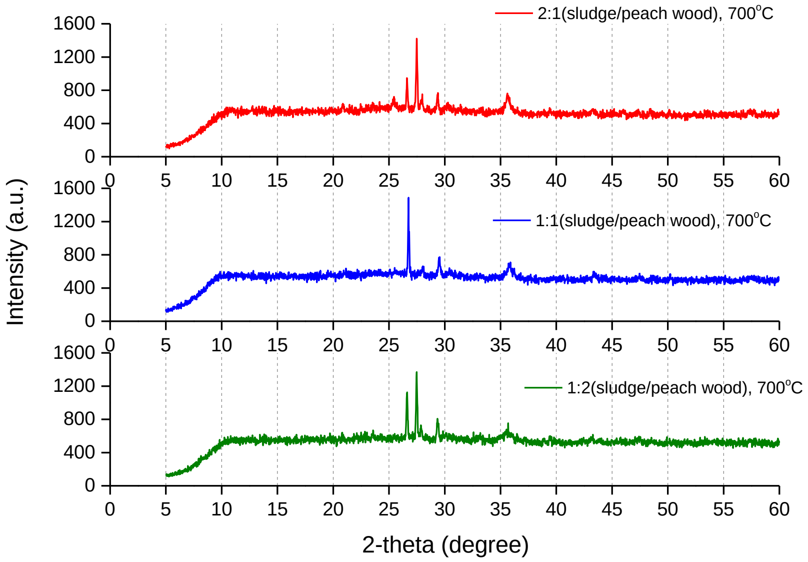

Figure S5.Powder X-ray diffraction diffractograms of biocharsat pyrolysis temperature of 700°C.
